# Therapeutic Colorectal Cancer Vaccines: Emerging Modalities and Translational Opportunities

**DOI:** 10.3390/vaccines13070689

**Published:** 2025-06-26

**Authors:** Palaniyandi Muthukutty, Hyun Young Woo, So Young Yoo

**Affiliations:** 1 Institute of Nanobio Convergence, Pusan National University, Busan 46241, Republic of Korea; pmkpalani@gmail.com; 2 Department of Internal Medicine, College of Medicine, Pusan National University, Busan 49241, Republic of Korea; who54@hanmail.net; 3 Medical Research Institute, Pusan National University Hospital, Busan 49241, Republic of Korea

**Keywords:** colorectal cancer, therapeutic vaccines, immunotherapy, neoantigens, tumor microenvironment, immune evasion, clinical translation, nanovaccines

## Abstract

Therapeutic vaccines offer a targeted approach to enhancing anti-tumor immunity with minimal systemic toxicity. Despite advancements in surgery, chemotherapy, radiation, and immunotherapy, colorectal cancer (CRC) remains a major clinical challenge, particularly due to the limited efficacy of immune checkpoint inhibitors outside the MSI-H subgroup. In this comprehensive review summarizes the emerging vaccine modalities for CRC, including peptide, nucleic acid, cell-based, vector-driven, and nanotechnology platforms. We discuss the barriers posed by tumor immune evasion and heterogeneity, and highlight innovative strategies designed to improve vaccine efficacy. Finally, we explore recent clinical developments and translational opportunities that position therapeutic vaccines as a promising component of future CRC immunotherapy.

## 1. Introduction

Colorectal cancer (CRC) is a heterogeneous malignancy driven by complex genetic and epigenetic alterations [1]. It is the third most common cancer worldwide and represents a significant public health burden in both developed and developing countries [2]. Each year, approximately 1.9 million new cases are diagnosed globally, with an estimated 930,000 deaths, underscoring its clinical importance [3]. CRC originates from the abnormal proliferation of glandular epithelial cells in the colon or rectum [4]. Multiple risk factors contribute to its development, including chronic inflammatory conditions such as ulcerative colitis and Crohn’s disease, family history, dietary habits, obesity, and physical inactivity [5,6]. The progression from benign adenomatous polyps to malignant carcinomas typically spans 10 to 15 years, emphasizing the critical importance of early detection and polypectomy in preventing cancer progression [7,8].

Despite advancements in screening programs, only about 40% of CRC cases are diagnosed at an early stage. Late-stage diagnosis significantly worsens the prognosis due to increased risks of systemic dissemination and metastasis [9]. Current standard treatments include surgical resection, chemotherapy, radiation therapy, targeted therapies, and immunotherapies [10]. While early-stage CRC patients exhibit relatively favorable five-year survival rates of approximately 65%, this rate drops dramatically to around 13% in cases of metastatic disease [11]. In addition, the emergence of chemotherapy-resistant tumor cells and treatment-induced toxicities further impair therapeutic outcomes and patient quality of life [12,13]. Tumor heterogeneity remains a major obstacle, contributing to therapy resistance and highly variable clinical responses [14].

In recent years, novel immunotherapeutic approaches—including immune checkpoint inhibitors, oncolytic virus therapies, adoptive T-cell therapies, and therapeutic cancer vaccines—have been actively explored as alternative or complementary strategies for CRC treatment [15]. Among these, therapeutic vaccines offer a particularly attractive approach by directly stimulating the host immune system to recognize and eliminate tumor cells, with minimal systemic toxicity [16]. Historically, early cancer vaccines utilized tumor lysates or whole inactivated tumor cells combined with immunostimulatory adjuvants such as Newcastle Disease Virus, Corynebacterium parvum, or Bacillus Calmette-Guérin [17].

Advances in cancer immunology have enabled the development of more sophisticated vaccine platforms targeting tumor-associated antigens (TAAs), such as carcinoembryonic antigen (CEA) and human chorionic gonadotropin β (hCGβ), and dendritic cell-based vaccines that present tumor antigens more effectively [18]. Dendritic cells (DCs) loaded with CRC antigens also represent a promising vaccine strategy [18]. Recombinant viral and bacterial vectors, as well as peptide- and nucleic acid-based vaccine approaches, have further expanded the arsenal of CRC vaccine strategies. To overcome immunosuppressive mechanisms within the tumor microenvironment (TME) and enhance vaccine efficacy, researchers are now incorporating adjuvant cytokine therapies, nanotechnology-based delivery systems, and personalized neoantigen targeting into vaccine designs [19].

This review aims to provide a comprehensive overview of the evolution and current status of colorectal cancer vaccines, highlighting the challenges posed by tumor-induced immune suppression, discussing innovative vaccine design strategies, and exploring translational opportunities to improve the clinical success of therapeutic vaccines in CRC.

## 2. Colorectal Cancer Development, Genetics and Classification—The Silent Pandemic

### 2.1. Development

The colon and rectum, forming the major components of the large intestine, play critical roles in nutrient absorption, water reabsorption, and maintaining the balance of the gut microbiota [20]. The colonic mucosa consists of numerous crypts lined by epithelial cells, with stem cells located at the crypt base that continuously regenerate the epithelial lining. This dynamic renewal process renders the colon particularly susceptible to diseases such as cancer [20]. Colorectal cancer arises from the malignant transformation of these epithelial cells, a process driven by the accumulation of genetic and epigenetic alterations over time [21].

Multiple risk factors contribute to CRC development, including advanced age, poor dietary habits, obesity, and chronic inflammatory bowel diseases such as ulcerative colitis and Crohn’s disease [22,23]. Although the precise early events of carcinogenesis remain incompletely understood, it is well established that the dysregulation of epithelial renewal initiates tumor formation [24]. The more common ‘classic’ or traditional pathway involves the development of adenomatous polyps (adenomas) that can progress to adenocarcinomas. An alternate pathway involves serrated polyps and their progression to serrated colorectal cancer [25]. Colorectal cancer is distinguished by its high mutational burden, driven by extensive somatic mutations across different CRC subtypes [7]. These genetic alterations result in the hyperproliferation of epithelial cells, the formation of benign polyps, and their progression into invasive carcinomas through distinct molecular pathways, including microsatellite instability (MSI), chromosomal instability (CIN), and the serrated neoplasia pathway [26].

Additionally, the incidence of CRC in younger people has been become increasingly significant [27]. Recent studies report an epidemiological shift in CRC incidence: in the United States, the rate among individuals over 50 has declined by 3.1% annually, whereas the incidence of early-onset CRC (eoCRC) has increased by 1.4% annually [28]. At this rate, eoCRC is projected to become a major public health issue by 2030, with incidence rates rising by 10.9% for colon and 22.9% for rectal cancers, respectively [27]. The prevalence of eoCRC is especially pronounced in high-income countries such as the United States, the United Kingdom, Australia, and New Zealand, with a particularly alarming rise observed in South Korea and Hong Kong [29]. These trends underscore the need for earlier screening protocols and preventive strategies to mitigate the burden of eoCRC [30,31].

**Figure 1:**
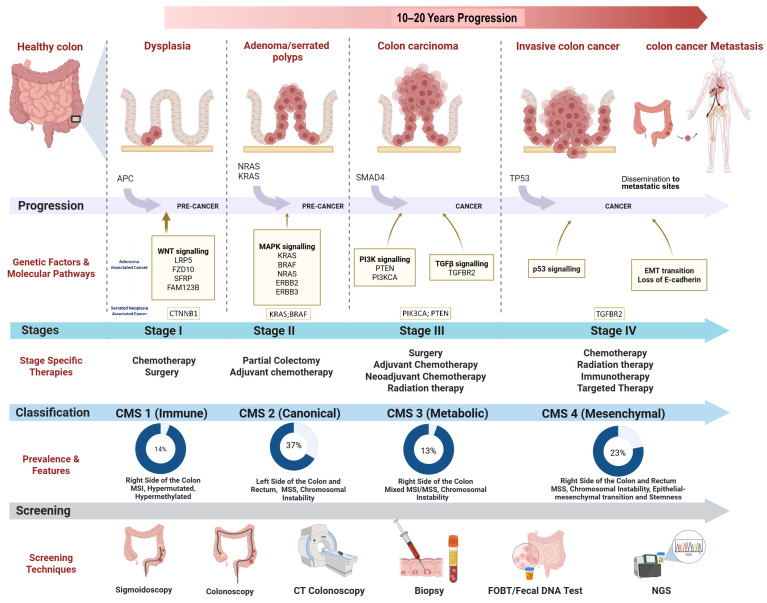
Progression, Classification, Screening of Colorectal Cancer (CRC). The development of colorectal cancer progresses through four stages: dysplasia, adenoma, carcinoma, and metastasis, driven by a variety of genetic and molecular pathways. Depending on the disease stage and severity, specific treatment approaches are recommended for CRC patients. Classification of CRC into Consensus Molecular Subtypes (CMS) provides valuable clinical prognostic and predictive information to guide therapy decisions. Advances in screening technologies have greatly improved the precision of CRC detection and treatment planning [25]. (Created in Biorender; Palaniyandi (2025) https://app.biorender.com/i-67fc6e5ec8be8eb93162a9c2-fig1crc (accessed on 25 March 2025)).

Clinically, CRC progresses through a series of well-defined stages (Stage 0 to IV). Stage 0 represents intraepithelial neoplasia or early dysplastic changes, Stage I involves invasion into the muscularis propria, Stage II is characterized by invasion through the serosa without nodal involvement, Stage III denotes the tumor further invades through muscularis propria into subserosa, where lymph node metastasis may be present or not, and Stage IV indicates distant organ metastasis (Figure 1). Early-stage patients (Stages I and II) demonstrate relatively favorable cure rates, ranging from 80 to 95% and 55 to 80%, respectively, whereas advanced-stage disease is associated with significantly worse survival outcomes [32,33]. Alarmingly, only about 40% of CRC cases are diagnosed at early stages [33].

### 2.2. Genetics

Colorectal carcinogenesis involves multiple processes, including aberrant cell proliferation, impaired differentiation, resistance to apoptosis, tissue invasion, and distant metastasis [34]. While hereditary factors contribute to a subset of cases, the majority of CRCs develop sporadically through a gradual accumulation of genetic mutations via the adenoma–carcinoma sequence over several decades [35]. Genetic mutations affecting key signaling pathways such as Wnt/β-catenin, p53, TGF-β, and EGFR are pivotal drivers of CRC initiation and progression [36]. Based on origin and genetic background, CRC is broadly classified into three major types [4,37]:

- Sporadic CRC (~60–80%): Occurs in individuals without a hereditary predisposition.
- Familial CRC (~20–40%): Associated with a family history but lacking identifiable high-penetrance mutations.
- Hereditary CRC: Includes familial adenomatous polyposis (FAP) and Lynch syndrome (hereditary nonpolyposis colorectal cancer, HNPCC).

Germline mutations in genes such as APC, DNA mismatch repair (MMR) genes, KRAS, and TP53 disrupt normal cellular regulation and are strongly implicated in CRC development [36]. Among these, APC mutations are particularly notable as early initiating events, detected in approximately 70% of sporadic colorectal adenomas [38]. The transition from adenoma to carcinoma typically requires additional mutations in genes such as KRAS, TP53, and SMAD4. Two major mechanisms of genomic instability underlie CRC development: microsatellite instability (MSI) and chromosomal instability (CIN). Mutations in APC have been associated with upregulated mismatch repair pathways, decreased expression of immune checkpoint molecules, lower MSI prevalence, and reduced tumor mutational burden (TMB).

In a small subset of sporadic CRCs, oncogenesis is driven by BRAF activation, DNA repair gene inactivation, and MSI. Several hereditary syndromes associated with autosomal dominant inheritance also contribute to increased CRC risk. These include familial adenomatous polyposis (FAP), Gardner syndrome, Lynch syndrome (HNPCC), Cowden syndrome, Turcot syndrome, juvenile polyposis syndrome, Peutz–Jeghers syndrome, and MUTYH-associated polyposis. Moreover, even in the absence of a defined syndrome, a positive family history of CRC substantially increases disease risk [38,39].

Based on molecular profiling, CRC can be classified into two principal subtypes: microsatellite stable (MSS) and microsatellite instability-high (MSI-H) cancers. MSS CRC accounts for approximately 85% of cases, whereas MSI-H CRC constitutes about 15% [37,40]. MSI-H tumors are generally associated with a better prognosis, diagnosis at a younger age, female predominance, and localization in the proximal colon. Conversely, MSS tumors are more frequently observed in older individuals, males, and are often located in the distal colon. MSI-H CRC is characterized by defective mismatch repair (dMMR), a higher tumor mutational burden, and a distinct molecular signature, in contrast to the proficient mismatch repair (pMMR) observed in MSS tumors [37,40].

The epithelial–mesenchymal transition (EMT) represents a critical phase in the later stages of colorectal cancer (CRC) progression, during which the disease acquires a more aggressive, invasive, and metastatic phenotype. During EMT, epithelial cells lose their polarity and intercellular adhesion properties—hallmarks of epithelial tissue—and instead adopt a fibroblast-like morphology with enhanced migratory and invasive capabilities, allowing them to infiltrate surrounding tissues [41]. The induction of EMT is primarily triggered by specific growth factors, notably transforming growth factor-beta (TGF-β) [42]. As cells undergo EMT, there is a downregulation of epithelial markers such as E-cadherin and zona occludens-1, and a concurrent upregulation of mesenchymal markers including vimentin and N-cadherin. Moreover, the loss of E-cadherin function, along with activation of the Wnt/β-catenin signaling pathway, are considered major drivers of EMT in CRC [43].

### 2.3. Classification

The genome integrity of CRCs is a crucial characteristic that distinguishes those that react effectively to immunotherapy from the ones that do not. This is established by assessing the ability of various CRC subtypes to do DNA repair, which determines the degree and kind of genomic instability they exhibit. Large-scale transcriptomic analyses have identified four consensus molecular subtypes (CMS) of CRC, each defined by distinct gene expression profiles [44,45] (Table 1):

The immunological subtype, CMS1, accounts for approximately 14% of colorectal cancers (CRCs), with microsatellite instability (MSI) observed in 76% of CMS1 tumors. In contrast, the canonical subtype, CMS2, represents about 37% of CRCs, yet only 2% of CMS2 tumors exhibit MSI. The metabolic subtype, CMS3, comprises approximately 13% of CRCs, while the mesenchymal subtype, CMS4, accounts for about 23% [45]. Microsatellite-stable (MSS) tumors are predominant among CMS3 and CMS4 subtypes, representing 84% and 94% of these groups, respectively. Each CMS subtype is characterized by a distinct clinical phenotype and genetic signature, offering valuable insights into tumor biology and serving as a foundation for future clinical classification and the development of subtype-specific therapeutic strategies [45]. Moreover, each CMS subtype exhibits unique histopathological features that are closely correlated with clinical outcomes and disease progression [37].

CMS categorization for CRC has proven to be a useful prognostic marker and predictor of therapy effectiveness. Deficient MMR (dMMR) results in high MSI, a hypermutable phenotype that is typically linked to successful immunotherapy responses. Many mutations in dMMR-MSI-H CRCs result in a high number of aberrant proteins that can behave as neoantigens, boosting the likelihood of tumor-infiltrating lymphocytes (TIL) recognition. Patients with these hypermutable cancers have a higher chance of survival and consistently respond to immunotherapies because they naturally consist of a large number of CD8+ TILs. Additionally, there is evidence that a high Tumor Mutation Burden (TMB) and an abundance of neoantigens are often not enough to trigger antitumor immunity. According to these classifications, the generation of neoantigens and other tumor-specific characteristics, several of which are genetically determined, influence how well cancer responds to immunotherapies and cancer vaccines.

## 3. CRC and Gut Microbes—The Microbiome Factor

The human gut microbiota plays a crucial role in maintaining intestinal homeostasis and regulating immune responses. It comprises trillions of microorganisms, including bacteria, archaea, viruses, and fungi, which interact symbiotically with the host [46]. The microbiota performs diverse functions, including the synthesis of vital metabolites, prevention of pathogen invasion, and regulation of bacterial proliferation to inhibit the growth of harmful species that could disrupt the local environment. As the digestive tract progresses from the stomach to the colon, the number and diversity of microbes increase substantially. Significant inter-individual variation exists in microbiota composition, influenced by a range of external environmental factors such as diet, exposure to chemicals, and the use of antibiotics and other medications.

The gastrointestinal tract serves as a critical interface between the host immune system and the intestinal microbiota. This metabolically active microbial community interacts intimately with both epithelial and stromal cells, playing a central role in maintaining both intestinal and systemic health. The composition and function of the gut microbiome are influenced not only by environmental factors but also by host genetics. Host–microbe interactions modulate a broad array of genes involved in innate and adaptive immunity, including those regulating adhesion molecules and epithelial barrier integrity. Recently, increasing attention has focused on the gut microbiome’s role in shaping cancer immunity and influencing responses to therapy [46,47,48].

The microbiome contributes to cancer immunity through multiple direct and indirect mechanisms that can either promote or suppress tumor growth and metastasis. These include modulation of systemic immune function, enhancement of immune surveillance, and regulation of inflammatory responses. In CRC, these dynamics are further complicated by the tumor’s influence on the immune environment. Tumors modify the composition of the gut microbiota through the expression of neoantigens and the development of immune evasion strategies. The tumor neoantigen burden plays a significant role in shaping the host immune response.

Compared to healthy individuals, patients with CRC exhibit a marked reduction in bacterial diversity and richness within the intestinal mucosa and fecal samples. Furthermore, significant shifts in specific bacterial groups have been observed, which may impact the mucosal immune response [49,50]. These alterations often involve a decrease in beneficial butyrate-producing bacteria and an increase in pro-inflammatory opportunistic pathogens, contributing to intestinal dysbiosis and, ultimately, tumor development. Notably, differences in the microbiota profiles between patients with early-stage (advanced adenoma) and advanced-stage CRC suggest that microbiome alterations may also influence tumor progression.

Several bacterial taxa, including Bacteroides fragilis, Fusobacterium nucleatum, Enterococcaceae, Campylobacter, Peptostreptococus, Enterococus faecalis, Escherichia coli, Shigella, and Streptococcus gallolyticus, are significantly enriched in CRC patients. In contrast, beneficial genera such as Faecalibacterium, Blautia, Clostridium, Bifidobacterium, and Roseburia are markedly decreased. Early dysbiosis characterized by higher levels of pro-inflammatory cytokines, and increased abundance of F. nucleatum has been associated with adenoma development and CRC progression [51,52,53].

In addition to bacteria, the gut microbiome includes viruses and fungi, which also play roles in CRC. Studies have shown that tumor tissues harbor a greater viral DNA load compared to healthy tissues. Research has investigated the potential contributions of viral infections—such as those caused by human papillomaviruses, human polyomaviruses, and human herpesviruses—to CRC risk [54]. It has been demonstrated that fecal virome profiles can differentiate individuals with early-stage or advanced CRC from healthy controls and may even predict CRC status [55].

Chronic inflammation is a well-established contributor to carcinogenesis, with estimates suggesting that persistent inflammation precedes approximately 20% of all cancers [56]. Dysbiosis of the gut microbiota, accompanied by increased intestinal permeability, is closely linked to colonic inflammation, potentially promoting CRC development and progression [57]. When intestinal permeability increases, bacterial components such as lipopolysaccharides (LPS) from Gram-negative bacteria can translocate into the host, triggering immune activation, cytokine release, and chronic inflammatory responses that favor tumorigenesis [58].

Beyond the microbiota itself, microbial metabolites also influence CRC development. Compounds such as β-glucuronidase, secondary bile acids, and short-chain fatty acids (SCFAs) produced by the gut microbiota interact with epithelial cells at the mucosal interface to modulate immune responses and disease risk [59]. Some metabolites, including polyamines and byproducts of protein fermentation, have pro-carcinogenic effects [60]. Polyamines—aliphatic amines essential for normal cell proliferation—are often dysregulated in cancer, particularly CRC, with aberrant polyamine metabolism contributing to tumor growth [61,62].

Anaerobic gut bacteria ferment dietary fibers to generate SCFAs such as butyrate, propionate, and acetate. These SCFAs are critical for maintaining colonic mucosal health, regulating local immune responses, and preserving intestinal barrier integrity [63]. Metabolomic studies have revealed notable disruptions in SCFA metabolism within CRC tissues compared to adjacent normal mucosa [64]. Through direct interactions with enterocytes, cellular metabolic regulation, and modulation of immune responses, the gut microbiome profoundly influences colorectal carcinogenesis [65].

## 4. CRC Screening and Surveillance—Evolving Innovations

Colorectal cancer (CRC) remains one of the leading causes of cancer-related deaths worldwide, despite advancements in screening technologies and early detection programs [66]. Even in high-income countries, the incidence, prevalence, and mortality rates of CRC remain substantially high, although some improvements have been achieved through enhanced screening and treatment efforts [67]. Alarmingly, the global prevalence of CRC is projected to nearly double by 2035, largely driven by an aging population and with the greatest increases expected in less developed regions [68]. The risk of developing CRC is influenced by both genetic predisposition and environmental factors. Germline mutations not only increase the likelihood of early-onset CRC but may also predispose individuals to other types of cancers [69]. Therefore, the implementation of effective surveillance and prevention strategies is essential for reducing morbidity and mortality, particularly through the early identification of individuals with hereditary colorectal cancer (CRC) [70]. Additionally, individuals with inflammatory bowel diseases, such as Crohn’s disease and ulcerative colitis, face an increased risk of developing CRC as they age [71]. Numerous studies have identified family history, chronic inflammation, dietary patterns, and lifestyle factors as significant risk contributors to CRC [72].

Screening for CRC enables the early detection of neoplastic and precancerous lesions, including adenomas and sessile serrated lesions [73]. Currently, approximately 60–70% of CRCs are diagnosed at an advanced stage. It is estimated that routine annual screening of healthy individuals could reduce overall CRC mortality by up to 60% while increasing the five-year survival rate from 65% to approximately 73% [23,74]. Screening of asymptomatic, average-risk individuals is the most efficient strategy for reducing CRC incidence and related deaths in the general population. In CRC, screening is particularly effective given the slow progression of the adenoma–carcinoma sequence. Although the exact timeline remains uncertain, existing evidence suggests that the transition from an early adenoma to an established carcinoma typically spans at least ten years, offering a substantial window for preventive interventions [75].

Moreover, the removal of colorectal adenomas during endoscopic procedures significantly reduces the risk of CRC development, and early detection of CRC dramatically improves patient survival rates [76]. Effective surveillance strategies include monitoring high-risk groups such as individuals with inflammatory bowel disease, those with hereditary CRC syndromes, individuals with a family history indicative of genetic susceptibility despite the absence of identifiable mutations, and patients with phenotypic characteristics suggestive of elevated CRC risk [73,77]. Screening is recommended for individuals aged 50 years and older, with current guidelines advocating regular testing due to its high efficacy and minimal adverse effects. Colonoscopy not only detects lesions but also enables the immediate removal of precancerous polyps, integrating diagnosis with prevention. Despite these efforts, only approximately 40% of CRCs are diagnosed at a localized stage. This is concerning, as the five-year survival rate drops precipitously from 90% to 13% once metastasis occurs. The American College of Gastroenterology (ACG) 2021, the U.S. Preventive Services Task Force (USPSTF) 2021, and the American Cancer Society (ACS) 2018 guidelines recommend CRC screening for individuals aged 50–75 years [78].

A variety of methods are available for CRC screening, including genetic testing for hereditary mutations, colonoscopy, flexible sigmoidoscopy, esophagogastroduodenoscopy, colon capsule endoscopy, computed tomographic (CT) colonography, and noninvasive stool-based tests such as the fecal immunochemical test (FIT), multitarget fecal DNA testing, plasma SEPT9 gene assay, and fecal occult blood test (FOBT). Among these, colonoscopy remains the most effective, demonstrating greater efficacy than sigmoidoscopy in preventing CRC—preventing approximately 12 cases and four deaths per 100,000 person-years [73,79]. Patients who are unable to tolerate sedation or colonoscopy may find that computed tomographic (CT) is a helpful alternative with 3D imaging of the colon with polyps and added advantage of low radiation risks [80,81]. Also, recent technological improvements have led to the introduction of capsule endoscopy, were a small pill like device takes pictures as it travels through the gastrointestinal tract [82]. Although there was no discernible difference in specificity, capsule endoscopy was shown to have greater sensitivity than CT [83]. FIT’s greater sensitivity makes it the most popular stool-based screening test [84]. It enables the detection of lower gastrointestinal bleeding associated with a variety of lesions, including precancerous ones, while not requiring dietary restrictions or as many stool samples as FOBT [85,86]. A novel technique using a liquid biopsy examines circulating tumor cells, circulating tumor DNA, exosomes, circulating tumor-derived endothelial cells, or protein molecules to find tumor-related markers [87]. Lofton-Day developed the plasma SEPT9 methylation assay in 2008 [88]. This latest developed test shows good sensitivity and specificity of the screening tests and received approval by USFDA [89,90]. Those who are susceptible or have a family history of colorectal cancer should undergo periodical screening by various techniques based on the patient’s clinical evaluation, comorbidities and the risk to benefit ratio can produce favorable clinical outcome to the CRC patients.

Recent advancements in artificial intelligence (AI) have further enhanced gastrointestinal endoscopy. Computer-aided detection (CADe) technologies have improved adenoma detection rates during colonoscopy, reducing the miss rate for clinically significant lesions by 15–20% [91]. Commercially available AI-assisted systems for adenoma detection include ENDO-AID (Olympus Corporation, Tokyo, Japan), EndoBRAIN-EYE (Cybernet Systems Corporation, Tokyo, Japan), GI Genius (Medtronic, Dublin, Ireland), and EndoScreener (Wision A.I., Shanghai, China). Continued advancements in these technologies are expected to further improve the sensitivity of colonoscopy, particularly in detecting advanced neoplastic lesions. Overall, significant strides have been made in CRC screening, contributing to improved survival outcomes. However, future efforts must focus on optimizing the effectiveness of preventive strategies and ensuring broader access to screening technologies [92,93].

## 5. Tumor Antigens Used in Colorectal Cancer Vaccine Development—The Source

One of the first steps in developing a colorectal cancer (CRC) vaccine is the identification of appropriate tumor antigens. Tumor antigens are broadly categorized into tumor-associated antigens (TAAs) and tumor-specific antigens (TSAs), commonly referred to as neoantigens. TAAs are proteins that are overexpressed in tumor cells compared to normal cells, whereas TSAs are uniquely expressed by cancer cells and are absent from normal tissues [94]. Significant advancements in cancer vaccine development have been made over the past decade. Following the U.S. Food and Drug Administration’s (FDA) approval of Sipuleucel-T in 2010, interest and research in cancer vaccines have expanded substantially. Several novel cancer vaccines have since been developed for CRC. Despite numerous efforts, identifying optimal tumor antigens in most malignant neoplasms has proven challenging [9]. This difficulty largely stems from the extremely low immunogenicity of many human cancers. Consequently, additional immune stimulation is required when designing therapeutic vaccines that utilize unmodified tumor cells [95,96].

Selecting the appropriate antigen is critical for the success of immunotherapeutic strategies, particularly in cancer vaccine development. TAAs are proteins that are typically overexpressed in tumor cells compared to normal tissues, enabling them to trigger T cell recognition and anti-tumor immune responses. Notable TAAs frequently targeted in CRC immunotherapy include carcinoembryonic antigen (CEA), melanoma-associated antigen (MAGE), epidermal growth factor receptor (EGFR), mucin-1 (MUC1), Wilms tumor 1 protein (WT1), survivin, squamous cell carcinoma antigen recognized by T-cells 3 (SART3), transmembrane 4 superfamily member 5 protein (TM4SF5), mitotic centromere-associated kinesin (MCAK), ring finger protein 43 (RNF43), guanylyl cyclase C (GUCY2C), translocase of outer mitochondrial membrane 34 (TOMM34), vascular endothelial growth factor receptors 1 and 2 (VEGFR1, VEGFR2), and insulin-like growth factor–II mRNA-binding protein 3 (IMP-3, also known as KOC1) [10].

Tumor-specific antigens (TSAs) or neoantigens arise from DNA mutations within cancer cells that lead to the production of altered proteins. TSAs are generated through various types of mutations, including frameshift mutations, gene fusions, insertions/deletions (indels), non-synonymous point mutations, and splicing mutations. In CRC clinical studies, vaccines targeting frameshift peptides derived from TGFBRII and CDX2, as well as mutant forms of KRAS, AIM2, TAF1B, and HT001, have been investigated. Furthermore, genes such as APC, KMT2D, ARID1A, SOX9, RNF43, TCF7L2, ZFP36L2, and TP53 have been identified as sources of neoantigens created through frameshift mutations that produce out-of-frame sequences [97,98,99]. Although the majority of neoantigens are either non-immunogenic or exhibit very low immunogenicity, recent studies demonstrating the ability of neoantigen-based vaccines to elicit robust immune responses have mitigated concerns regarding their therapeutic potential. Consequently, active efforts are underway to develop personalized cancer vaccines targeting these neoantigens [100].

The selection of neoantigens for personalized vaccine development is typically prioritized based on their prevalence among CRC patients [101]. Accordingly, predefined libraries of peptide, RNA, or DNA vaccines targeting frequently occurring frameshift mutations in CRC could provide an “off-the-shelf” solution, paving a clear path forward for CRC treatment and prevention strategies [102].

## 6. Therapeutic Vaccines for CRC—The Savior

**Figure 2:**
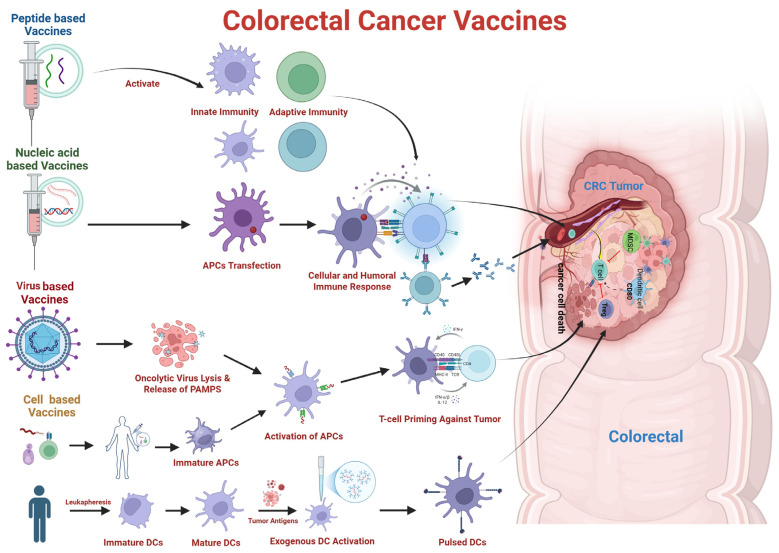
Modalities for Colorectal Cancer Vaccines. A schematic representation illustrating the various strategies employed in the development of colorectal cancer vaccines and their mechanisms for activating the immune system. These modalities promote immune-mediated tumor neutralization through diverse immunological pathways. (Created in Biorender; Palaniyandi (2025) https://app.biorender.com/i-67fcc6f74856de77a515ab6d-fig3-crc (accessed on 27 March 2025).

Therapeutic cancer vaccines offer promising alternatives to conventional immunotherapies for various cancer types. They aim to enhance anti-tumor immunity and promote tumor regression or eradication, typically with minimal side effects. Furthermore, therapeutic vaccines help prevent inappropriate immune reactions that are not specifically targeted against tumors [103]. Cancer vaccines are capable of generating robust immune responses against specific antigens, thereby increasing tumor-infiltrating lymphocyte (TIL) infiltration and inducing cytotoxic effects against cancer cells expressing those antigens. Upon vaccine administration, tumor-specific antigens are captured by antigen-presenting cells (APCs), particularly dendritic cells (DCs), initiating the cancer-immunity cycle. Antigen-loaded DCs migrate to draining lymph nodes, where they prime T cells, leading to the generation of both effector and memory T cells. Activated tumor-specific effector T cells then traffic to the tumor microenvironment via the bloodstream, releasing cytotoxic cytokines and directly killing tumor cells (Figure 2).

Following tumor cell death, numerous tumor-associated antigens and damage-associated molecular patterns (DAMPs) are released. These molecules are subsequently captured, processed, and presented by APCs, thereby stimulating polyclonal T-cell responses and reinforcing anti-tumor immunity. Therapeutic cancer vaccines, thus, contribute to perpetuating the cancer-immunity cycle by shifting the immune balance from tolerance to active tumor rejection. Notably, compared to conventional therapies, cancer vaccines generally exhibit favorable safety profiles and low dose-limiting toxicities [104].

### 6.1. Peptide/Protein Based Vaccines

Peptide and protein-based vaccines are designed to stimulate immune responses against specific tumor antigens. These vaccines typically contain either entire proteins or protein fragments derived from tumor-specific proteins and are administered along with adjuvants to enhance immunogenicity. Peptide vaccines present immunogenic epitopes derived from TAAs or TSAs to major histocompatibility complex (MHC) class I or II molecules on the surfaces of antigen-presenting cells (APCs), leading to the activation of T cells and the induction of long-lasting antigen-specific immune memory [105]. Several TAAs have been identified and targeted via MHC class I pathways in colorectal cancer, including p53, β-human chorionic gonadotropin (β-hCG), SART3, carcinoembryonic antigen (CEA), mucin-1 (MUC1), and Survivin-2B [106]. However, despite extensive research, most peptide vaccines targeting these antigens have failed to demonstrate substantial survival benefits in clinical settings.

To overcome these limitations, novel peptide vaccines targeting multiple epitopes have been developed, and numerous clinical investigations are currently underway. For instance, a peptide vaccine cocktail comprising three peptides demonstrated safety and immunogenicity in HLA-A24-positive colorectal cancer patients. Another recently developed peptide vaccine, PolyPEPI1018, incorporates 12 unique epitopes derived from seven conserved tumor antigens commonly expressed in metastatic CRC (mCRC). In a clinical study, PolyPEPI1018 was administered to 11 patients and was found to be safe and well tolerated, eliciting increased CD4+ and CD8+ T cell responses against at least three different antigens. Further studies are needed to fully establish its safety and efficacy before commercialization [107].

ATP128 is another promising vaccine currently under investigation. It is a chimeric recombinant protein vaccine produced using the KISIMA™ platform, consisting of three major components: a multi-antigenic domain (Mad), a proprietary Toll-like receptor agonist (TLRag) peptide with self-adjuvanting properties, and a cell-penetrating peptide (CPP) for efficient antigen delivery. The Mad includes three TAAs relevant to CRC—Survivin, achaete-scute complex homolog 2 (ASCL2), and CEA. ATP128 activates dendritic cells (DCs) through TLR2 and TLR4 signaling pathways, resulting in cytokine secretion and upregulation of costimulatory molecules essential for T cell priming. It is currently being evaluated in a Phase Ib clinical trial (KISIMA-01; NCT04046445) for patients with Stage IV MSS/pMMR CRC [108].

Peptide-based vaccines offer several key advantages, including the ability to elicit highly specific anti-tumor immune responses and relatively low manufacturing and storage costs [109,110]. Nevertheless, they are hampered by significant challenges such as low intrinsic immunogenicity, tumor-mediated immune evasion, and antigenic escape that can lead to tumor recurrence [111]. Ongoing clinical trials are increasingly focusing on multi-antigen peptide vaccines combined with potent adjuvants to overcome these obstacles [105]. Notably, the focus has shifted in recent years to tumor analysis (whole genome or exome sequencing), individualized cancer vaccine testing, and the selection and production of optimum neoantigens due to the challenges in obtaining the required outcomes using peptide/protein vaccines [112].

### 6.2. Nucleic Acid Based Vaccines

DNA vaccines are composed of plasmids encoding one or more tumor antigens. These plasmids can be transcribed and translated to antigens are presented then by both MHC class I and II molecules and simultaneously activate innate immune responses through cytosolic receptors [113]. Among the various antigens explored in CRC DNA vaccine development, MYB, an oncoprotein aberrantly expressed in multiple cancers including CRC, has attracted significant interest. MYB promotes cell proliferation, inhibits differentiation, and enhances resistance to apoptosis, making it a promising immunotherapy target [114,115]. DNA vaccines targeting MYB have been designed to overcome self-tolerance by co-expressing fusion proteins and MHC tetanus peptides, thereby enhancing T cell activation. Preclinical studies using CRC transgenic mouse models have demonstrated that MYB-based DNA vaccines elicit strong protective and therapeutic effects [116]. Research into cancer DNA vaccines has continued to evolve, with the latest generation of DNA vaccines demonstrating broader immune responses in experimental models compared to earlier versions [117,118].

RNA-based vaccines, produced in vitro and encoding tumor-specific antigens, have also been extensively studied as both preventive and therapeutic agents [119,120]. Similarly to DNA vaccines, mRNA vaccines offer several additional advantages, including cytoplasmic expression without the need for nuclear entry, minimizing the risk of genomic integration. Upon internalization by target cells, mRNA is translated into tumor antigens, which are presented by APCs via MHC class I molecules to activate robust CD8+ cytotoxic T cell responses [121]. Recent studies have also demonstrated that adding specific signal peptides to mRNA sequences encoding tumor antigens can promote presentation via MHC class II molecules, thereby stimulating CD4+ T helper cell responses [122]. As a result, there is growing interest in mRNA vaccines as promising cancer immunotherapy strategies, and numerous early-phase clinical trials are currently investigating their potential in CRC [123]. mRNA-4650, developed by Moderna Therapeutics, is an mRNA-based cancer vaccine undergoing clinical testing for several malignancies, including CRC. In a Phase I/II trial, intramuscular administration of mRNA-4650 in CRC patients successfully induced CD8+ and CD4+ T cell responses against neoantigens, without significant adverse effects or tumor relapse [124].

Another candidate from Moderna, mRNA-5671 (V941), is a therapeutic cancer vaccine targeting KRAS-positive cancers, including CRC. Preclinical studies have demonstrated that mRNA-5671 significantly enhances CD8+ T cell responses both as monotherapy and in combination with pembrolizumab, an anti-PD-1 antibody. Additionally, mRNA-4157, another Moderna mRNA vaccine, encodes up to 34 distinct neoantigens and has been tested in combination with pembrolizumab. In patients with MSI-H CRC, mRNA-4157 was found to be safe, well tolerated, and capable of inducing neoantigen-specific CD8+ and CD4+ T cell responses, leading to both partial and complete tumor responses. Based on these encouraging findings, mRNA-4157 is now advancing to Phase II clinical trials [125]. Across multiple early-phase clinical and preclinical studies, mRNA-based cancer vaccines have shown promising evidence of anti-tumor immune activity in various solid tumors, including CRC [126,127]. Ongoing research aims to further validate the safety, immunogenicity, and therapeutic efficacy of these platforms.

The potential of nucleic acid cancer vaccines is being investigated in a number of past and current clinical investigations employing a variety of techniques. DNA and mRNA cancer vaccines has a great potential, but there are still a number of issues that need to be resolved. TAAs have historically been the focus of numerous DNA and mRNA cancer vaccines, but their therapeutic success has been limited. On the other hand, more recent strategies have focused on using TSAs that are more immunogenic. These include neoantigens and viral antigens, which are being investigated in a number of clinical trials at the moment. Next-generation DNA and mRNA cancer vaccines promise a new era of precision oncology, but their successful development will depend on the quick identification and validation of neoantigens [128]. Next concern is GMP-grade DNA/mRNA synthesis is a costly and time-consuming procedure. Although the development of personal DNA/mRNA cancer vaccines is a promising area, there are several obstacles in the way of its manufacturing, especially with regard to cost-effectiveness, Good Manufacturing Practices (GMP) compliances, and improving the stability of DNA/mRNA formulations. Because mRNA preservation usually necessitates extremely low temperatures, such −20 °C or −70 °C, the broad shipping of mRNA-based vaccines is severely restricted by this demand for cold chain transportation [129]. The development of precision oncology strategies and the fight against CRC ultimately depend on the successful integration of various approaches and the creation of customized vaccines.

### 6.3. Cancer Cell Based Vaccines

Cancer cell-based vaccines are designed to prime the immune system by using whole tumor cells or their lysates as sources of tumor antigens [130]. Depending on the origin of the cancer cells, these vaccines are classified as either autologous or allogeneic. Autologous vaccines, derived from a patient’s own tumor cells, offer personalized antigenic profiles, whereas allogeneic vaccines are derived from established tumor cell lines and are advantageous in terms of production scalability and broader applicability [131]. One of the most extensively studied CRC vaccines is OncoVax, which emerged in early-phase clinical trials in the 1980s. OncoVax combines autologous tumor cells with bacille Calmette-Guérin (BCG), a well-known immune adjuvant [100].

Although initial clinical trials using OncoVax in combination with surgical resection failed to show significant improvements in clinical outcomes, recent studies combining OncoVax with 5-fluorouracil (5-FU) and leucovorin have demonstrated its safety in CRC patients [132]. The allogeneic whole-cell vaccine GVAX has been genetically modified to secrete granulocyte-macrophage colony-stimulating factor (GM-CSF), thereby enhancing its immunogenic potential. In a Phase II trial involving patients with advanced pMMR CRC, GVAX exhibited modulatory effects on anti-tumor immune responses [133]. In a Phase I study, patients with metastatic CRC (mCRC) were vaccinated with irradiated allogeneic CRC cells combined with bystander cells engineered to produce GM-CSF. In addition, patients received a single low dose of cyclophosphamide to deplete regulatory T cells (Tregs), thereby enhancing vaccine efficacy. The combination was found to be well tolerated and capable of inducing anti-MUC1 antibody responses [134].

The pursuit of effective therapeutic cancer vaccines has focused on identifying antigens capable of activating cytotoxic T lymphocytes (CTLs) to recognize and destroy cancer cells. A key advance was the understanding that dendritic cells (DCs), as professional APCs, play a central role in T cell activation. Upon maturation, DCs migrate chemotactically from peripheral tissues to T cell-rich areas of lymph nodes while upregulating costimulatory molecules, cytokine secretion, and HLA–peptide complexes essential for efficient T cell priming [135].

Various protocols have been developed to generate mature DCs ex vivo, with one common method involving the isolation of CD14+ monocytes from peripheral blood, which are then differentiated into mature monocyte-derived DCs (moDCs) capable of presenting tumor antigens [136]. MoDCs pulsed with autologous tumor lysates have been used in CRC patients to ensure presentation of neoantigens and other tumor antigen classes [137]. Multiple clinical trials have confirmed the safety of moDC vaccines and their ability to elicit tumor-specific T cell responses associated with prolonged survival [136]. However, challenges remain, particularly regarding the availability of tumor material for vaccine preparation. To address this, moDCs have also been pulsed with synthetic peptides or mRNA encoding tumor-associated antigens, lysates from allogeneic cell lines, or well-characterized neoantigens such as frameshifted TGF-β receptor II and caspase variants.

Although moDCs are commonly used, it has been argued that traditional DC subsets might possess superior intrinsic abilities to stimulate robust CTL responses compared to moDCs [138]. In one study, DCs mobilized using an FMS-like tyrosine kinase 3 ligand (Flt3L) and pulsed with tailored carcinoembryonic antigen (CEA) peptides improved clinical responses in CRC patients [139]. Furthermore, the use of CD34+ hematopoietic stem cells or induced pluripotent stem cells (iPSCs) to generate DC subsets ex vivo offers new avenues for enhancing vaccine potency [140]. Given the time, labor, and cost challenges associated with ex vivo DC production, various in situ targeting approaches have been explored in CRC, utilizing vaccine platforms based on peptides, mRNA, viral vectors, bacterial vectors, or irradiated autologous/allogeneic CRC cells combined with adjuvants. These approaches aim to deliver antigens and maturation stimuli directly to DCs within the body [141,142,143,144,145]. It is now widely recognized that to fully realize the therapeutic potential of cancer vaccines, they must be combined with strategies that support the function of activated T cells within the immunosuppressive tumor microenvironment (TME). Advancements in targeted vaccine delivery to specific DC subsets are expected to further improve clinical outcomes in CRC.

### 6.4. Vector-Based Vaccine

Vector-based vaccines leverage biological vectors such as viral vectors, live-attenuated bacteria, and yeasts to deliver tumor antigen transgenes and elicit potent immune responses. These vectors inherently express pathogen-associated molecular patterns (PAMPs), which are recognized by host pattern recognition receptors (PRRs), activating innate immunity and facilitating the induction of adaptive anti-tumor responses. Recombinant viral vectors are engineered to express immunogenic tumor-associated antigens (TAAs) and simultaneously evoke innate inflammatory signals, enhancing the specificity and potency of anti-tumor immunity.

Among viral antigen platforms, lentiviruses, retroviruses, poxviruses, and adenoviruses have been the most widely utilized [146,147]. Virus-based vaccines for CRC have been a focus of intensive research for several decades, and numerous clinical trials have been conducted in the past 10 years. Adenovirus-derived vaccines such as Ad5 [E1-, E2b-]-CEA(6D) and Ad5-hGCC-PADRE, as well as vaccines derived from alphaviruses (e.g., PANVAC, TroVax, AVX701), poxviruses, and modified vaccinia Ankara vectors, have all undergone Phase I–II trials. However, none have yet progressed to Phase III trials or been approved for clinical use [148]. The limited clinical success of viral vector-based CRC vaccines is likely attributable to their insufficient ability to induce durable and robust anti-tumor immunity when used as monotherapies, underscoring the need for combination approaches with other therapeutic agents.

In a Phase II clinical trial conducted by Redman et al., 26 patients with untreated microsatellite-stable (MSS) metastatic CRC were randomized to receive either the standard-of-care (SOC) regimen (avelumab, mFOLFOX6, and bevacizumab) alone or the SOC regimen combined with the Ad5 [E1-, E2b-]-CEA (AdCEA) vaccine [149]. Patients in the SOC plus immunotherapy (SOC + IO) group experienced a slightly prolonged progression-free survival (PFS) of 10.1 months compared to 8.8 months in the SOC-only group. Furthermore, heterogeneous CD4+/CD8+ T cell responses to cascade antigens (e.g., MUC1, brachyury) were enhanced in 8 of 11 patients in the SOC + IO group compared to only 1 of 8 in the SOC group. Currently, multiple clinical trials are underway investigating virus-based CRC vaccines in combination with other immunotherapeutic agents, aiming to enhance anti-tumor immunity and overcome the limitations observed in monotherapy settings.

### 6.5. Live-Attenuated Bacteria and Yeast Vaccines

Live-attenuated bacterial vaccines have demonstrated the capacity to induce robust intratumoral inflammatory responses, humoral immunity, and cell-mediated immunity. Due to their inherent immunogenicity, bacteria harbor numerous PAMPs, such as cross-linked glycan chains, which are recognized by innate immune receptors, including Toll-like receptors (TLRs). Activation of these receptors leads to the recruitment of antigen-presenting cells (APCs) and the secretion of pro-inflammatory cytokines. As a result, live-attenuated bacterial vectors can effectively stimulate strong and durable immune responses against tumor neoantigens. Extensive research has been conducted to develop cancer vaccines using bacteria genetically engineered to deliver tumor neoantigen-encoding genes. These engineered bacterial vaccines have demonstrated superior safety, specificity, and therapeutic efficacy compared to conventional tumor vaccines.

Several live-attenuated bacterial vaccination platforms for CRC have been developed in preclinical studies. However, only a few have advanced into clinical trials. Among them, Listeria monocytogenes-based platforms have shown the most promise. For instance, a Phase I clinical trial (NCT03189030) evaluated the safety and tolerability of a customized live-attenuated, double-deleted (pLADD) L. monocytogenes-based immunotherapy for metastatic CRC [150]. Additionally, yeast-based vaccines have emerged as another promising approach. Heat-killed Saccharomyces cerevisiae vectors encoding tumor-associated antigens such as carcinoembryonic antigen (CEA) or tumor-specific antigens (TSA) have been studied. Vaccines like GI-6207 and GI-4000, derived from heat-killed, genetically modified S. cerevisiae, have demonstrated safety and immunogenicity in Phase I clinical trials.

Yeast-based immunotherapy has been shown to successfully elicit protective T cell immune responses against various mutated and overexpressed tumor neoantigens. Their distinct biological properties—including non-replicative nature, strong immunogenicity, and favorable biocompatibility—make yeast platforms highly attractive for therapeutic protein production and vaccine development. Specifically, GI-6207, encoding CEA, has demonstrated good tolerability and no dose-limiting toxicities in patients with metastatic CEA-expressing carcinomas, including CRC [151]. Similarly, GI-4000, encoding mutated forms of Ras, has induced strong cytotoxic T-cell responses against Ras mutations and is being evaluated in combination therapies in a Phase Ib/II trial for metastatic CRC (NCT03563157) [152].

Overall, live-attenuated bacterial and yeast-based vaccines represent a highly promising future direction for CRC immunotherapy. Their ability to deliver tumor antigens efficiently and stimulate potent immune responses suggests they may eventually become standard components of cancer vaccine strategies in clinical settings.

### 6.6. Neoantigen Vaccines

Tumor cells accumulate a variety of genetic alterations, some of which give rise to neoantigens—novel peptide sequences perceived as foreign by the host immune system. These neoantigens result from mutations, frameshift errors, gene fusions, or alterations in noncoding genomic regions. These neoantigens can be presented by antigen presenting cells to high avidity T cell receptors of tumor-specific T cells, allowing tumor cell destruction [153]. Recent advances in bioinformatics, sequencing technologies, and multi-omics platforms have significantly expanded the ability to identify neoantigens. High-throughput genome sequencing combined with computational peptidome analysis has enabled the prediction of neoantigen immunogenicity and HLA-binding affinity [154]. Due to the relatively high mutational burden in colorectal cancer (CRC)—particularly in the microsatellite instability-high (MSI-H) subgroup—multiple MHC class I ligands are generated. Frequent, fixed-pattern mutations in microsatellite regions contribute to the production of abundant neoantigens in MSI-H tumors [155].

Tumor mutation burden (TMB) has been identified as highest in MSI/dMMR malignancies. The elevated TMB in these cancers is associated with an increased infiltration of neoantigen-specific T cells and a better response to immune checkpoint blockade therapies or chemotherapy. TMB serves as an indicator of the number of T cells and the presence of neoantigens within the tumor microenvironment [156]. Common oncogenic driver mutations, including those in KRAS, TP53, and DNA repair genes such as POLE, generate neoantigens that have been shown to be immunogenic. Consequently, therapeutic vaccination targeting these neoantigens and adoptive transfer of neoantigen-specific T cells are promising strategies under active preclinical and clinical investigation [157].

A Phase I clinical trial evaluated the immunogenicity and safety of frameshift peptide neoantigens derived from mutations in AIM2, HT001, and TAF1B in 22 patients with dMMR CRC [158,159,160]. The results demonstrated that vaccination with a single frameshift peptide was capable of inducing both humoral and cellular immune responses in all patients without significant vaccine-related toxicities. Nous-209, an off-the-shelf cancer vaccine encoding multiple frameshift peptides, is currently undergoing evaluation in combination with pembrolizumab for the first-line treatment of patients with dMMR/MSI-H locally advanced, unresectable, or metastatic CRC (NCT04041310) [161,162].

Current approaches to neoantigen targeting include vaccines aimed at shared neoantigens across multiple patients, as well as highly personalized vaccines tailored to each individual’s tumor-specific mutations. Since most tumor mutations are unique to each patient, personalized neoantigen vaccines require individualized tumor tissue sequencing, design, validation, and manufacturing. Although this process remains time-consuming and costly, resolving these challenges is critical to making neoantigen-based vaccines more accessible for widespread clinical application in CRC.

### 6.7. Nanovaccines in Colorectal Cancer

One of the most promising strategies to enhance the efficacy of cancer immunotherapy involves the development of nanovaccines, which integrate biomedical nanotechnology with immunological principles. Nanovaccines exhibit multiple advantages, including multivalent delivery to lymphoid tissues and efficient phagocytosis by antigen-presenting cells (APCs). The structural basis of nanovaccines lies in the encapsulation of antigens and immunological adjuvants within nanocarriers, which may include liposomes, polymers, inorganic materials, or biomimetic particles [163]. Nanoscale modifications improve antigen delivery by enhancing homing, retention, and accumulation in lymph nodes, thereby increasing the efficiency of antigen presentation and detection by APCs. Due to their pseudo-adjuvant properties, nanoparticles can potentiate immune responses and facilitate the effective delivery of immunogens. Their distinctive physical and chemical features—including pH sensitivity, magnetic, optical, and electrical properties—enable real-time tracking, targeted release, and multi-modal therapeutic integration, making nanovaccines highly versatile tools for cancer immunotherapy [164].

Nanoparticle albumin-bound (nab)-paclitaxel, a nanoformulation of paclitaxel attached to albumin nanoparticles, has been evaluated in metastatic CRC. In a study by Overman et al., nab-paclitaxel was combined with fluorouracil, leucovorin, and oxaliplatin (FOLFOX) as a first-line treatment for metastatic CRC. Compared to standard FOLFOX therapy, the combination exhibited an improved overall response rate (ORR) and a favorable safety profile, suggesting that nab-paclitaxel can enhance the efficacy of conventional chemotherapy [165].

Polymeric nanocarriers with varying compositions and cargo retention properties are well-suited for vaccine vector design. Some polymers target endosomal compartments via pH- or enzyme-responsive mechanisms, while others function as in situ cancer vaccines [166]. For example, BaNV is a self-assembling nanovaccine formulated using maleimide-functionalized poly(ethylene oxide)-block-poly(D,L-lactic acid) (MAL-PEG-b-PLA) micelles. It encapsulates a neoantigen peptide (Adpgk) along with two synergistic adjuvants. When administered in combination with anti-PD-1 therapy, BaNV enhanced PD-1 receptor sensitization and achieved a 70% complete remission rate of neoantigen-specific tumors in murine models [167]. Phospholipid-based liposomes, capable of encapsulating both hydrophilic and lipophilic agents, have also shown utility in nanovaccine design. In a Phase II study, patients with mCRC who underwent R0/R1 resection of colorectal liver metastases were treated with L-BLP25 (tecemotide), which contains a 25-amino-acid MUC1-derived peptide. Median relapse-free survival (RFS) and overall survival (OS) in the tecemotide group were 6.1 months and 62.8 months, respectively. Although not statistically significant compared to other trials (CELIM and FIRE-3), patients undergoing secondary resection showed improved OS outcomes [168,169].

Inorganic nanoparticles, including gold, iron, and silica, offer additional opportunities due to their unique physical characteristics. Porous silicon microparticles allow controlled release of tumor antigens and adjuvants, inhibiting CRC growth in murine models [170]. A notable example is CCM@(PSiNPs@Au), a hybrid nanovaccine combining photothermal therapy and cancer vaccination using cancer cell membranes and gold/silicon nanocomposites [171]. Another multifunctional platform, Cu_2_O@CaCO_3_@HA, demonstrated synergistic CRC-targeted effects through photothermal, photodynamic, chemo-dynamic, and calcium overload-mediated mechanisms in the CT26 murine model [172].

ImmTher, a lipophilic disaccharide tripeptide derivative of muramyl dipeptide (MDP), encapsulated in liposomes, overcame MDP’s limitations of hydrophilicity and rapid clearance. In Phase I trials, ImmTher demonstrated anti-tumor activity against CRC liver and lung metastases [173,174]. In another study, monophosphoryl lipid A (MPL-A) was used in a liposomal emulsion formulation containing recombinant baculovirus-derived KSA (Ep-CAM). This induced both humoral and Th1-associated cellular immune responses and was well tolerated. When combined with GM-CSF, the formulation enhanced specific T helper cell activity in CRC patients [175].

Exosomes are biologically derived extracellular vesicles secreted by a variety of cell types, including bacteria, mammalian cells, and cancer cells. As naturally occurring nanoparticles, exosomes are emerging as promising carriers for next-generation nanovaccines. They are capable of encapsulating a wide range of biomolecules, such as proteins, mRNAs, microRNAs, and DNA [176,177]. Owing to their excellent biocompatibility and non-replicative nature, exosomes derived from both bacterial and mammalian sources are considered attractive platforms for immunotherapeutic delivery [178]. A Phase I clinical study involving 40 patients with mCRC evaluated exosomes derived from ascitic fluid (Aex) combined with GM-CSF. These exosomes carried tumor-associated CEA and multiple immune regulatory markers. Patients positive for HLA-A0201 and CEA were randomized to receive either Aex alone or Aex with GM-CSF. Both treatments were safe and well tolerated, but the Aex plus GM-CSF group exhibited stronger anti-tumor cytotoxic T cell responses, suggesting its potential as a safe and effective therapeutic vaccine [179].

Importantly, most nanovaccines appear to exert maximal efficacy when used in combination with other therapies, underscoring the importance of multimodal treatment strategies. However, only a limited number of nanovaccines have successfully progressed from laboratory research into clinical application.

### 6.8. Other Vaccines in Development for Colorectal Cancer

The primary issue in developing a vaccine against a pathogen is determining which antigen will best trigger a robust and protective immune response for an infectious pathogen. Whereas for a vaccination to be effective in case of cancers, the tumor microenvironment must be taken into account in addition to tumor antigens and HLA variability. The creation of a universal vaccine against colorectal cancer is made more challenging by the fact that each patient has different antigens, HLA, and TME. Several attempts are made to create vaccine based on the existing immuno-technologies. One of the emerging techniques is that to use B-cell as vaccine. Although B-cells are primarily recognized for their capacity to generate antibodies, they can also enhance the immune response in other ways, such as by acting as antigen-presenting cells. B-cells’ power to absorb, internalize, and digest antigens is typically correlated with their ability to present antigens; this ability is dependent on surface immunoglobulins that are specific to that antigen [180]. Several studies have demonstrated the efficacy and functionality of using B-cells as a vaccine candidate. In one of the works, they have demonstrated that tumor regression may result from naïve B-cells enriched with tumor-derived autophagosomes that selectively capture TSAs after being triggered by CD40 or TLR in E.G7 murine thymoma models [181]. In another study, Oxley et al., demonstrated that using tumor cell lysates as stimulating antigens to stimulate B-cells could be an efficient technique for developing a B-cell-based cellular vaccination against tumors [180].

Another evolving concept of vaccine is using of virus-like particles (VLP) as antigens, as they can evoke the immune response and are able to induce cytotoxic T cell and antibody responses [182]. For CRC, a mouse model of colorectal cancer has been created using rabbit hemorrhagic disease virus (RHDV) VLPs that contain surviving, and murine topoisomerase IIα (topIIα) as epitopes generated from CRC TAAs showed improved overall survival and tumor growth delay [183]. With the understanding that the main hurdle in vaccine development is still identifying the target antigen, this suggests that B-cell vaccines and VLPs can be used in the future to more effectively deliver the potential remedies.

In the fight against cancer, cancer vaccines offer a promising approach that increases the immune system’s capacity to identify and destroy cancer cells while utilizing its capability to stop tumor development, recurrence, or metastasis. Despite the tremendous opportunity for cancer vaccination, a number of restrictions prevent these vaccines from being widely used and from working as effectively as they could. Each vaccine has its own limitations in effectiveness of delivery, as the cancer is a highly complicated disease. Effectiveness is complicated by tumor genetic diversity since certain cells may not produce the targeted antigens, which results in less-than-ideal immune responses. In certain cases, vaccine formulations are often complicated by the requirement for adjuvants or immune-stimulating chemicals. Cancer vaccine therapy tends to be more effective in individuals with a robust immune system, low tumor burden, and a higher risk of recurrence. In this respect, a strong immune response activity against tumor cells may be produced by combining vaccinations with other immunotherapy techniques. It is hoped that in the coming years, developments in bioinformatics, sequencing technologies, and possibly machine learning and artificial intelligence will enable the removal of these obstacles and the simple identification of patient-specific antigens and HLA specificity from the standpoint of advanced vaccines.

## 7. Colorectal Cancer Vaccines in Clinical Trials

Reducing mortality and improving therapeutic outcomes in colorectal cancer (CRC) requires the development and clinical validation of effective vaccines. However, the substantial heterogeneity of CRC poses a major challenge, necessitating the integration of vaccines into multimodal treatment regimens to overcome therapeutic barriers. Some new cancer vaccines that have demonstrated encouraging preclinical results in the treatment of colorectal cancer are presented in this section. These vaccines include protein-based vaccines, cell-based vaccines, emerging nucleic acid-based vaccines, oncolytic viruses, and neoantigen and vector-based vaccines. Autologous DC-based vaccines utilizing tumor antigens have been extensively studied in both preclinical and clinical settings. Among them, the CEA RNA-pulsed DC vaccine has demonstrated safety and therapeutic efficacy in patients with resected liver metastases from colon cancer [184]. In another study, they used the influenza virus, injected intratumorally to an early-stage CRC patient before surgery and showed there was better CD8+ T cell infiltration into the tumor with transcription analysis showing increased cell killing activities and reduced neutrophil-related genes [185]. Detailed review of these clinical trials and the significance of these clinical trials are discussed elaborately elsewhere [186]. Several CRC vaccine candidates are currently under clinical investigation. Table 2 summarizes representative vaccine strategies registered on ClinicalTrials.gov, including their immunotherapeutic approaches, clinical phases, routes of administration (ROA), combination therapies, trial statuses, NCT numbers, and references.

## 8. Other Treatment Strategies Against CRC—The Alternatives

Historically, the primary treatment modalities for cancer patients included surgery and chemotherapy, followed by radiotherapy, targeted therapies, and immunotherapies (Table 3). Despite these advancements, the prognosis for CRC patients with metastatic disease has generally remained poor. Improvements in both primary and adjuvant therapies have extended survival for CRC patients. An integrated assessment of patient-related factors (e.g., prognosis, comorbidities) and tumor-specific characteristics (e.g., tumor location, metastasis, biomarker involvement) is crucial for determining the optimal course of treatment. Approximately 20% of CRC cases develop metastases, and nearly a quarter are diagnosed at an advanced stage, where curative surgical control is often difficult to achieve, leading to tumor-related mortality [206].

### 8.1. Chemotherapy

Current chemotherapy regimens include monotherapies such as 5-fluorouracil (5-FU) and combination therapies involving capecitabine (CAP), irinotecan (IRI), and oxaliplatin (OX), which aim to overcome treatment resistance and stabilize tumors before or after surgery [207]. For the past two decades, standard CRC treatment has centered on 5-FU combined with oxaliplatin and/or irinotecan [208]. In patients unresponsive to irinotecan-based regimens, second-line therapies such as FOLFOX (5-FU, leucovorin, oxaliplatin) or CAPOX (capecitabine, oxaliplatin) are employed [209]. Conversely, patients resistant to oxaliplatin may be treated with irinotecan monotherapy or FOLFIRI (5-FU, leucovorin, irinotecan) [210].

Treatment duration varies but typically spans up to six months. Chemotherapy-related adverse effects in CRC include leukopenia, thrombocytopenia, diarrhea, polyneuropathy, severe nausea (hyperemesis), hepatotoxicity, renal dysfunction, and general health deterioration. Elderly patients and those with comorbidities are particularly susceptible to severe toxicities [13].

### 8.2. Immunotherapy

Immunotherapy enhances the body’s natural defense mechanisms to target and eradicate cancer cells [211]. In CRC, immunotherapeutic strategies include adoptive cell therapy, immune checkpoint inhibitor (ICI) therapy, monoclonal antibody therapy, complement inhibition, cytokine therapy, and cancer vaccines [212,213]. Immune checkpoints regulate T cell activation to prevent autoimmunity, while ICIs block these inhibitory signals, enhancing anti-tumor responses [214]. Renewed interest in immunotherapy for metastatic CRC (mCRC) followed the discovery of key immune checkpoints such as CTLA-4 and PD-1 and the development of ICIs like nivolumab and pembrolizumab [215].

PD-1 acts as a key negative regulator of T cell activation, and its ligand PD-L1 (CD274) facilitates T cell apoptosis or functional inactivation. PD-1/PD-L1 inhibitors restore T cell activity by blocking these interactions. However, therapeutic responses to ICIs have been largely restricted to patients with MSI-H or dMMR tumors [216]. The FDA approved nivolumab and pembrolizumab for dMMR/MSI-H CRC in 2014, and both agents have shown durable efficacy [217]. Additional FDA-approved monoclonal antibodies for CRC treatment include cetuximab, ipilimumab, ramucirumab, panitumumab, and bevacizumab [217]. Immunotherapy is often combined with chemotherapy, radiotherapy, and ICIs to maximize therapeutic benefits and minimize adverse effects [218].

### 8.3. Targeted Therapy

Targeted therapies have significantly improved survival outcomes in CRC. Agents such as the anti-EGFR monoclonal antibody cetuximab and the anti-angiogenic agent bevacizumab have shown considerable efficacy [207]. Targeted strategies under clinical development for CRC include those directed against EGFR, VEGF/VEGFR, c-MET, HGF, TGF-β, Wnt/β-catenin, and IGF/IGF-1R pathways [207]. Cetuximab, approved in 2004, was the first targeted agent for CRC [219]. Since then, numerous additional targeted therapies have been developed and commercialized, with many more undergoing preclinical and clinical evaluation [220].

### 8.4. Adoptive Cell Therapy

Adoptive cell therapy (ACT) involves extracting autologous T cells from patients, expanding and modifying them ex vivo, and reinfusing them to boost anti-tumor immunity [221]. Initially, tumor-infiltrating lymphocytes (TILs) were used, but peripheral blood mononuclear cells are now preferred due to technical challenges associated with TIL isolation [222].

Genetically engineered T cells expressing tumor antigen-specific receptors offer a way to target diverse malignancies [223,224]. Recent advances allow the in vitro generation of activated killer cells, including NK, αβ T cells, and γδ T cells [225]. Ex vivo-differentiated T cells from sentinel lymph nodes have shown promise in Phase I/II clinical studies, significantly extending median survival compared to controls [226]. Nonetheless, ACT faces challenges such as target-dependent toxicity, long preparation times, and high costs [227].

New therapeutic strategies, including gene editing, cytokine therapies, and complement inhibition, are under development to enhance ACT efficacy [228].

### 8.5. CAR-T Cell Therapy

Chimeric antigen receptor (CAR)-T cell therapy is a form of ACT where T cells are genetically modified to express tumor-specific receptors [229]. Unlike TCRs, CARs can recognize tumor antigens independently of HLA presentation [230]. CARs typically incorporate a single-chain variable fragment (scFv) linked to intracellular signaling domains such as CD3ζ [231], and they are introduced into T cells using lentiviral or gammaretroviral vectors [232]. After ex vivo expansion, CAR-T cells are reinfused into patients, often following lymphodepletion therapy. CAR-T cell therapies are currently under clinical investigation in CRC, showing promising early results [233].

Certain antigens, including human epidermal growth factor receptor 2 (HER2), NK group 2 member D ligand (NKG2DL), epithelial cell adhesion molecule (EpCAM), mesothelin (MSLN), MUC-1, and CD133, have been approved for use in clinical trials due to their high expression levels in CRC patients [234]. Detailed review of these antigens and their different phases in the clinical trials are discussed elaborately elsewhere [235]. Preclinical models and early-stage clinical trials have shown a range of potential CAR-T treatment approaches for colorectal cancer, underscoring the extensive research being conducted to identify the most effective targets or combinations of state-of-the-art checkpoint inhibitors and monoclonal antibodies [236,237].

### 8.6. Oncolytic Virus Therapy

Oncolytic viruses (OVs) represent a novel class of cancer therapeutics that enhance tumor lysis by selectively replicating within tumor cells and stimulating host anti-tumor immune responses. A wide range of DNA and RNA viruses have been explored as OVs, with poxviruses, reoviruses, herpes simplex viruses, and adenoviruses being the most commonly employed in current clinical cancer research [238,239]. In addition to their tumor-lytic properties, the therapeutic effects of OVs involve the coordinated modulation of the immune system and the TME. Furthermore, viral genome engineering techniques allow the insertion of transgenes into the OV genome, enabling tumors to be immunomodulated through the expression of therapeutic genes encoded by the virus. [239]. The first FDA-approved OV, talimogene laherparepvec (T-VEC), paved the way for OV-based immunotherapy [240]. In preclinical studies, GM-CSF-expressing vaccinia viruses, such as JX-594 (Pexa-Vec), demonstrated potent anti-tumor activity against CRC peritoneal metastases by restoring local immunity [241]. Similarly, modified vaccinia virus VG9-IL-24 exhibited enhanced CRC killing and immune activation [242]. Other engineered adenoviral vectors, including SPDD-UG and rAd.DCN.GM, have also shown promise in CRC models by delivering therapeutic genes such as GAS5, SNORD44, and GM-CSF [242,243].

Arming OVs with immune checkpoint inhibitors, such as PD-1/PD-L1 antibodies, has further enhanced anti-tumor responses [244]. When the PD-L1 inhibitor and GM-CSF were co-expressed by the modified oncolytic vaccinia virus (VV-iPDL1/GM), they observed that intra-tumor injection of these OVs stimulated immune cells and encouraged tumor infiltration of neoantigen-specific T cells leading to the elimination of treated tumor and the distant tumors [245]. A vesicular stomatitis virus (VSV) carrying an M51R mutation has been genetically modified to express a single-chain variable fragment (scFv) derived from the human IgG1 antibody avelumab, which targets PD-L1. In preclinical studies, this modified VSV demonstrated significant suppression of tumor growth in mouse models [246]. These findings highlight that recombinant oncolytic viruses (OVs) engineered to express PD-1/PD-L1-targeting antibodies represent a promising new strategy for cancer immunotherapy.

Currently, multiple clinical trials have demonstrated the therapeutic potential of OVs when combined with immune checkpoint inhibitors (ICIs). While ICIs alone have not shown substantial clinical benefit in the treatment of microsatellite stable/proficient mismatch repair (MSS/pMMR) metastatic colorectal cancer (mCRC), emerging clinical data suggest that combining OVs with ICIs may offer new hope for patients with pMMR/MSS or MSI-Low mCRC [247].

## 9. Adjuvants, Dosage and Route of Administration—Value Addition

To maximize immune responses and enhance the antitumor efficacy of cancer vaccines, preclinical and clinical trials must carefully optimize vaccine dosage, selection and use of adjuvants, routes of administration, and vaccination schedules. Additionally, a thorough understanding of the mechanisms underlying T cell activation, subset differentiation, and the development of long-term memory is crucial for advancing cancer vaccine applications.

### 9.1. Adjuvants

Adjuvants are agents that boost immune responses by enhancing antigen presentation by APCs. Their primary role in antitumor therapy is to stimulate, sustain, and extend the immune response throughout the treatment period [248]. Adjuvants are particularly critical when tumor cells are insufficiently immunogenic to mount a robust immune defense. Their use can increase vaccine efficacy by promoting the activation of NK cells and cytotoxic CD8+ T cells without negatively impacting humoral immune responses [249]. Common adjuvants employed in cancer vaccines include cytokines, galectin inhibitors, and immune checkpoint inhibitors. Among these, cytokines such as GM-CSF have been widely utilized. GM-CSF promotes the production of interleukin-1 (IL-1), tumor necrosis factor (TNF), and interleukin-6 (IL-6), while enhancing T and B cell activation and fostering strong immune stimulation [250]. Galectin inhibitors and monoclonal antibodies can also serve as adjuvants, often showing synergistic effects when combined with cancer vaccines. In one study, patients were treated with pembrolizumab (anti-PD-1) or ipilimumab (anti-CTLA-4) in combination with the galectin inhibitor GR-MD-02, resulting in more effective therapeutic responses compared to monotherapy [251]. Another notable adjuvant system is TRICOM (Triad of Costimulatory Molecules), which incorporates three key costimulatory molecules expressed by APCs. TRICOM enhances the proliferation and functional avidity of antigen-specific memory CD8+ T cells and has been extensively investigated in clinical trials, particularly in viral vector-based vaccines [252].

Additionally, co-encapsulation strategies involving cancer antigens and phosphorylated adjuvants within lipid-calcium-phosphate nanoparticles have shown promise for targeting CRC liver metastases. These nanoparticles carried the peptide antigen p-AH1-A5 and adjuvants such as 5′pppdsRNA, CpG, and 2′3′cGAMP, leading to more effective suppression of tumor progression compared to conventional controls [253]. Similarly, a DNA vaccine using CpG combined with interleukin-2 (IL-2) demonstrated enhanced cytotoxicity against CRC metastases in preclinical models [254]. Other frequently used adjuvants include polyinosinic-polycytidylic acid (poly I:C), CpG oligonucleotides, and IFN-γ, all of which can enhance the effectiveness of cancer vaccines under various conditions [255,256,257].

### 9.2. Dosage and Route of Administration

Determining the optimal vaccine dosage is crucial, as subtherapeutic doses may fail to induce effective immune responses, whereas excessively high doses may trigger cytokine release syndrome, dose-limiting toxicities, or unwanted innate immune activation. Previous clinical studies have demonstrated that higher vaccine doses within a safe range enhance immune responses and antitumor efficacy. The route of vaccine administration is equally critical for maximizing immunogenicity, ensuring precise delivery, supporting large-scale manufacturing, and advancing clinical development [258]. Common administration routes for CRC vaccines include intramuscular, intravenous, subcutaneous, and intradermal injections, each with specific advantages and challenges [259].

Intramuscular injection typically involves the use of adjuvants to recruit APCs and stimulate local inflammation. In contrast, intravenous delivery must overcome barriers such as serum protein aggregation and rapid degradation, which can be mitigated by encapsulating nucleic acid vaccines within protective carriers [260]. Multiple-site vaccination strategies have shown promise in enhancing antigen-specific T cell responses by expanding antigen delivery across multiple lymphatic drainage areas, thus increasing overall vaccine efficacy [261].

Moreover, a detailed understanding of antigen kinetics can optimize vaccine performance. Studies have shown that antigen exposure over several days generates stronger antiviral immunity compared to single or multiple bolus doses [262]. Therefore, factors such as timing of administration, cumulative dosing, and prime–boost intervals must be carefully evaluated in clinical trial design.

Nevertheless, establishing consistent dose–response relationships remains challenging due to patient heterogeneity and the current lack of extensive vaccine-specific clinical data [263]. Thus, novel techniques for tracking the spatiotemporal kinetics of vaccines are urgently needed to refine immunization strategies and maximize therapeutic outcomes. [259].

## 10. Conclusions and Future Perspectives

The global incidence of colorectal cancer (CRC) is projected to rise over the coming decades due to population growth, aging, and the increasing prevalence of major lifestyle-related risk factors such as unhealthy diets, physical inactivity, and obesity. Currently, the rising incidence of eoCRC has been linked to lifestyle-related factors such as sedentary behavior, poor dietary habits, and obesity among younger populations. Additionally, individuals with a familial genetic predisposition should undergo regular screening as recommended by clinical guidelines. Enhanced awareness and education among primary healthcare providers could play a critical role in reducing the onset of eoCRC through timely identification and intervention [264,265]. Consequently, cancer control initiatives should be prioritized, with secondary prevention strategies playing a pivotal role in reducing the burden of CRC in an economically sustainable and efficient manner. Effective and widely implemented screening programs with high adherence rates have the potential to substantially decrease CRC mortality.

Significant progress has been made in the development of cancer vaccines for CRC. While the approval of therapeutic cancer vaccines such as Oncophage for kidney cancer and Provenge (sipuleucel-T) for prostate cancer marked important milestones, similar success has not yet been achieved in CRC, particularly in advanced disease stages. Despite ongoing clinical trials, no CRC vaccine candidate has demonstrated significant survival benefits in Phase III trials. One major challenge lies in the inability of vaccine-induced anti-tumor immunity to persist long enough to achieve meaningful improvements in patient survival. Several factors contribute to this limitation, including the lack of an ideal tumor antigen, adjuvant, vaccine platform, and delivery system. Furthermore, without addressing the complex immunosuppressive mechanisms within the tumor microenvironment (TME)—such as regulatory T cells (Tregs), myeloid-derived suppressor cells (MDSCs), and immunosuppressive molecules—the functional capacity of vaccine-induced CD8+ T cells is likely to be compromised.

It also remains uncertain whether preventive cancer vaccines targeting non-viral tumors like CRC are realistic goals or overly ambitious, especially given the relatively high efficacy of early CRC detection and treatment for precancerous lesions. To advance cancer care and research, governments should prioritize CRC awareness campaigns, ensure timely and equitable access to high-quality medical care, promote personalized medicine approaches, and facilitate patient enrollment in clinical trials. Greater investment in these areas will be crucial to achieving better outcomes for CRC patients. Importantly, recent advances in the identification of neoantigens and the development of nanovaccines are rapidly reshaping the field of cancer immunotherapy. These innovations offer renewed hope that CRC vaccines will soon become integral components of combination immunotherapy strategies, providing durable clinical benefits to patients with colorectal cancer.

## Figures and Tables

**Table 1:** Consensus Molecular Classification (CMS) of Colorectal Cancer.

Classification	Prevalence	Features
CMS 1 (Immune)	14%	MSI-H CIMP high; hypermutation strong immune activation and JAK-STAT signaling pathway activation
CMS 2 (Canonical)	37%	SCNA high; Wnt/MYC signaling activation
CMS 3 (Metabolic)	13%	Mixed MSI status, SCNA low, CIMP high; Metabolic dysregulation
CMS 4 (Mesenchymal)	23%	SCNA high; Stromal infiltration, TGF-β activation, angiogenesis

Consensus molecular classification (CMS); Janus kinase/signal transduction and transcription activation (JAK-STAT); Wingless-related integration site/myelocytomatosis oncogene (Wnt/MYC); CpG island methylator phenotype (CIMP); Somatic Copy Number Alterations (SCNA).

**Table 2:** CRC Vaccines Currently in Clinical Trials.

Vaccine Types	Type of Immunotherapy	Clinical Phase	ROA	Vaccination Strategy	Combination Therapies	Status	NCT Number	Ref.
**Cellular** **Vaccines**	Cancer Stem Cell Vaccine	I/II	i.v	Cancer Stem Cell Vaccine against Specific Antigen in Metastatic Adenocarcinoma of the Colorectal Cancer	NA	Completed	NCT02176746	[187]
**Combination Vaccine**	Combination Immunotherapy in Subjects with Advanced Small Bowel and Colorectal Cancers	II	s.c, i.v	Combination of vaccines and immunotherapy drugs can reduce the tumor for colorectal cancers	II) CEA/MUC1 Vaccines + M7824 + N-803 + NHSIL12 (Quadruple Therapy)	Completed	NCT04491955	[188]
					I) CEA/MUC1 Vaccines + M7824 + N-803 (Triple Therapy)			
**Combination Vaccine**	Mix Vaccine	I/II	s.c	Mix vaccine to small metastases of colorectal cancer	NA	Completed	NCT03357276	[189]
**Combination Vaccine**	GVAX Colon Vaccine (With Cyclophosphamide) and Pembrolizumab	II	i.d	For Patients with Mismatch Repair-Proficient (MMR-p) in Advanced Colorectal Cancer	Cyclophosphamide, Pembrolizumab	Completed	NCT02981524	[133]
**Combination Vaccine**	AlloStim^®^ Immunotherapy Alone and in Combination with Cryoablation as Third Line Therapy	II	i.t, i.v	Bioengineered allogeneic immune cells acts as adjuvant to modulate immune response and kill metastatic tumor cells	Cryoablation	Completed	NCT02380443	[190]
**Combination Vaccine**	SGI-110 in Combination with an Allogeneic Colon Cancer Cell Vaccine (GVAX) and Cyclophosphamide (CY)	I	i.d, i.v	Against Metastatic Colorectal Cancer (mCRC) as Maintenance Therapy	GM-CSF, Cyclophosphamide	Completed	NCT01966289	[191]
**DC Vaccine**	RNA-Pulsed DC vaccine	I/II	i.v	CEA RNA Pulsed patients’ dendritic cells are reinfused into the patient’s body	NA	Completed	NCT00003433	[184]
**DC Vaccine**	Labelled DC Vaccine	I	i.d	MRI-based Tracking of Alpha-type-1 DC Vaccines in Patients with Colorectal Cancer	NA	Completed	NCT01671592	[192]
**DC Vaccine**	Autologous Dendritic Cell Vaccine plus Avelumab	I/II	i.d	For Pre-treated Mismatch Repair-proficient (MSS-p) Metastatic Colorectal Cancer Patients	Avelumab	Completed	NCT03152565	[193]
**DC Vaccine**	CEA-loaded dendritic cell vaccine	I/II	i.d, i.v	Induction of Specific T Cell Responses in Colorectal Cancer Patients with Liver Metastases	oxaliplatin/capecitabine	Completed	NCT00228189	[194]
**Nucleic acid Vaccine**	V941(mRNA-5671/V941)	I	i.v, i.m	Participants with KRAS Mutant Colorectal Cancer	Pembrolizumab	Completed	NCT03948763	[195]
**Neoantigen Vaccine**	Cancer Vaccine Targeting Shared Neoantigens (GRT-C903 and GRT-R904)	I/II	s.c, i.v, i.m	Neoantigen in combination with checkpoint inhibitors to stimulate immune response against CRC patients	nivolumab	Completed	NCT03953235	[196]
					ipilimumab			
**Neoantigen Vaccine**	Neoadjuvant PalloV-CC	I	i.d	Autologous DC vaccine with silicate-capped yeast cell wall protein by ex vivo priming loaded with tumor lysate for colon cancer	NA	Completed	NCT03827967	[197]
**Neoantigen Vaccine**	Cancer Vaccine Targeting Shared Neoantigens (GRT-C901 and GRT-R902)	I/II	s.c, i.v, i.m	Vaccine regimen uses two vaccine vectors as a heterologous prime/boost approach (GRT-C901 first followed by GRT-R902) to stimulate an immune response.	nivolumab	Completed	NCT03639714	[198]
					ipilimumab			
**OV Vaccine**	Influenza Vaccine for Early Colorectal Cancer	I/II	i.t	Intratumoral application of an unattenuated influenza vaccine	Curative Surgery	Completed	NCT04591379	[185]
**OV Vaccine**	Recombinant Ad5 (CEA/MUC1/ Brachyury) Based Immunotherapy Vaccine	I	s.c	Novel adenovirus-based vaccine to elucidate antitumor cytolytic T-cell responses	NA	Completed	NCT03384316	[199]
**OV Vaccine**	Immunotherapy With CEA(6D) VRP Vaccine (AVX701)	I	i.m	Active Immunotherapy against patients with Stage III Colorectal Cancer		Completed	NCT01890213	[200]
**OV Vaccine**	5T4-MVA (TroVax)	II	i.m	Against patients undergoing surgical resection for colorectal liver metastases	NA	Completed	NCT00259844	[145]
**Peptide Vaccine**	IMA910 Plus GM-CSF	I/II	i.d	For advanced colorectal carcinoma patients	GM-CSF, Cyclophosphamide	Completed	NCT00785122	[201]
**Peptide Vaccine**	Colorectal Cancer Peptide Vaccine PolyPEPI1018	I	s.c	Used against proteins present on the surface of CRC tumor cells	TAS-102	Completed	NCT05130060	[202]
**Peptide Vaccine**	PolyPEPI1018 Vaccine and CDx	I/II	s.c	Add-on Immunotherapy to the Standard-of-Care Maintenance Therapy	Fluoropyrimidine/Bevacizumab maintenance therapy	Completed	NCT03391232	[107]
**Peptide Vaccine**	MUC1 Peptide-Poly-ICLC Adjuvant Vaccine	II	s.c	For Individuals with Advanced Colorectal Adenoma	NA	Completed	NCT00773097	[203]
**Peptide Vaccine**	Vaccine Therapy with or Without Interleukin-2	I/II	i.v, s.c	For Treatment of HLA A2.1 Positive Patients with Colorectal Cancer	NA	Completed	NCT00019591	[204]
**Vector based Vaccine**	V934/V935 hTERT Vaccination	I	i.m, i.d	Vaccination in Cancer Patients with Selected Solid Tumors	NA	Completed	NCT00753415	[205]
**Yeast based Vaccine**	Vaccine (GI-6207)	I	s.c	Vaccine for Metastatic CEA- Expressing Carcinoma	NA	Completed	NCT00924092	[151]

DC: Dendritic cell; OV: Oncolytic virus; GM-CSF: Granulocyte Macrophage-Colony Stimulating Factor; CEA: Carcinoembryonic Antigen; i.v: Intravenous; s.c: Subcutaneous; i.d: Intradermal; i.t: Intratumor; i.m: Intramuscular.

**Table 3:** Approved Treatment Regimen for CRC.

Name	Commercial Names	Target	Year of Commercialization	Treatment
Bevacizumab	Avastin	VEGF	2004	Metastatic colorectal cancer
Cetuximab	ERBITUX	EGFR	2004	Metastatic and Recurrent colorectal cancer
Panitumumab	Vectibix	EGFR	2006	Metastatic carcinoma of the colorectal cancer
Regorafenib	Stivarga	VEGFR2&3; TIE2	2012	Metastatic carcinoma of the colorectal cancer
Ziv-aflibercept	Zaltrap	VEGF and PlGF	2012	Metastatic carcinoma of the colorectal cancer
Ramucirumab	Cyramza	VEGFR2	2015	Metastatic carcinoma of the colorectal cancer
Nivolumab	Opdivo	PD-1	2017	Advanced MSI-H/dMMR colorectal cancer
Ipilimumab	Yervoy	CTLA-4	2018	MSI-H or dMMR colorectal cancer
Pembrolizumab	Keytruda	PD-1	2020	Advanced or unresectable MSI-Hor dMMR CRC cases
Dostarlimab	Jemperli	PD-1	2021	Recurrent or advanced dMMR solid tumors

VEGF: Vascular Endothelial Growth Factor; VEGFR: Vascular Endothelial Growth Factor Receptor; EGFR: epidermal growth factor receptor; PD-1: Programmed death receptor-1; CTLA-4: Cytotoxic T lymphocyte antigen 4; Placental growth factor (PlGF).

## Data Availability

The data of this study are available from the corresponding authors on reasonable request.
